# Supplementary material for: Volcanoes stunt nearby glaciers
Source: Nat Commun. 2025 Aug 29;16:8099. doi: 10.1038/s41467-025-63332-2 (PMC12397297; doi:10.1038/s41467-025-63332-2)
Supplement: Supplementary file 1 — Supplementary Information [file 41467_2025_63332_MOESM1_ESM.pdf]

Volcanoes stunt nearby glaciers

Supplementary material

Tryggvi Unnsteinsson[1*], Matteo Spagnolo[2], Brice R. Rea[1], Társilo Girona[3,4], Iestyn Barr[5], Donal Mullan[6]

[1]School of Geosciences, University of Aberdeen, Aberdeen, Scotland, United Kingdom.
[2]Department of Earth Sciences, University of Torino, Torino, Italy.
[3]Geophysical Institute, Alaska Volcano Observatory, University of Alaska Fairbanks, Fairbanks, Alaska, USA.
[4]Geosciences Barcelona (GEO3BCN-CSIC), Barcelona, Spain.
[5]Department of Natural Sciences, Manchester Metropolitan University, Manchester, England, United Kingdom.
[6]School of Natural and Built Environment, Queen's University Belfast, Belfast, Northern Ireland, United Kingdom.

*Corresponding author(s). E-mail(s): t.unnsteinsson.23@abdn.ac.uk;

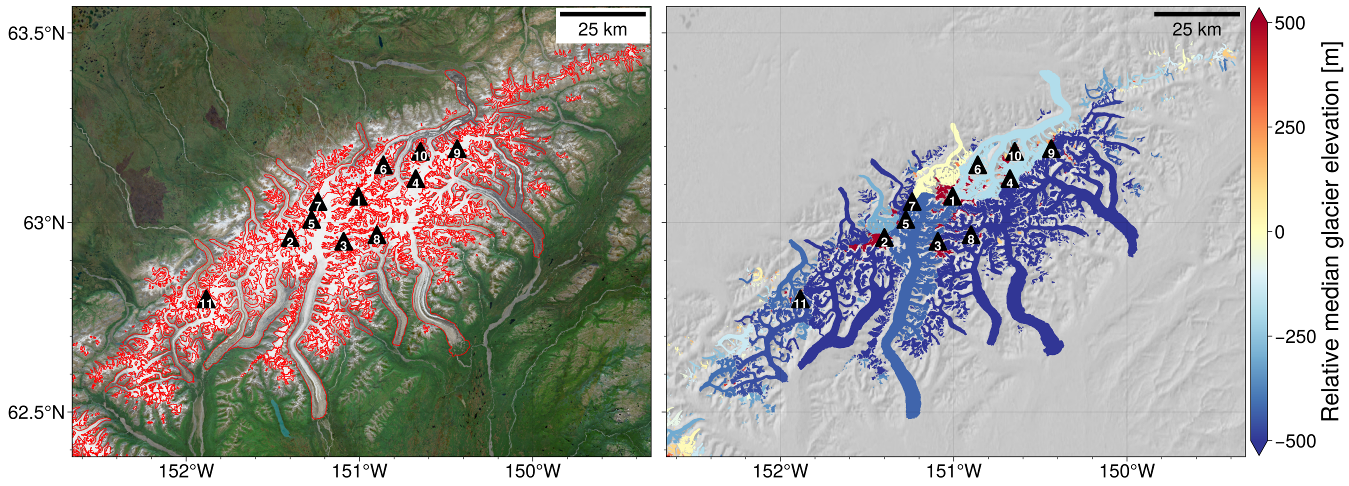

Fig. S1 The Central Alaska Range: **(left)** true colour imagery from modified Copernicus Sentinel data [2024] processed in Copernicus Browser, with the RGI glacier outlines in red and **(right)** the relative median glacier elevations. The black triangles denote some of the regions highest peaks: (1) Denali, (2) Mount Foraker, (3) Mount Hunter, (4) Mount Silverthrone, (5) Mount Crosson, (6) Mount Carpe, (7) Kahiltna Dome, (8) Mount Huntington, (9) Mount Mather, (10) Mount Brooks, and (11) Mount Russell. Median glacier elevations do not seem to be correlated with distance from high mountains. Several dry-calving glaciers can be seen as positive anomalies around prominent peaks, such as around (1). The base map is a shaded relief of the ASTER GDEM V003 [S1].

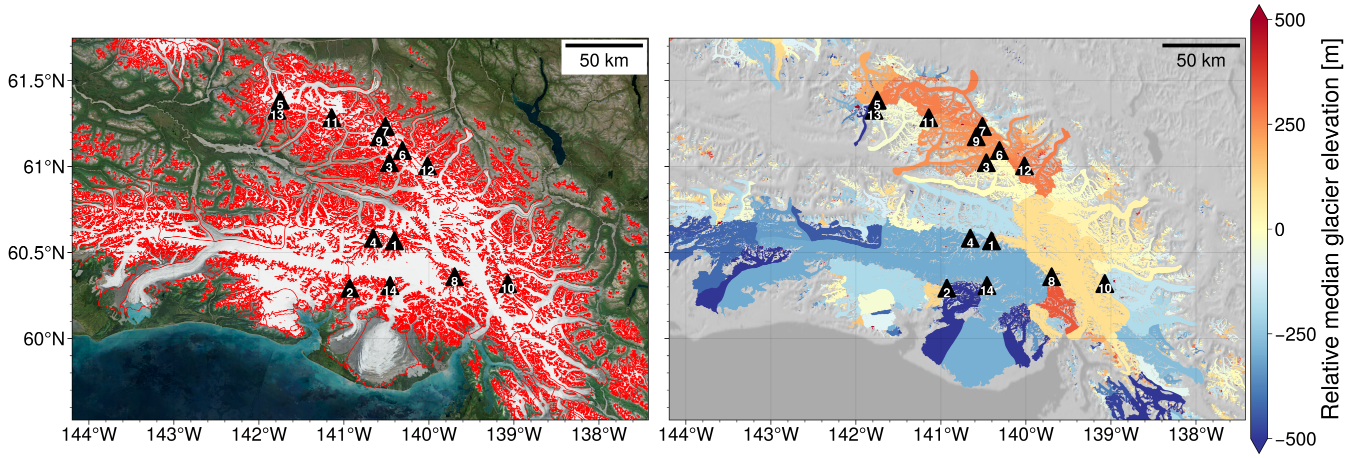

Fig. S2 The Saint Elias Mountain Range: **(left)** true colour imagery from modified Copernicus Sentinel data [2024] processed in Copernicus Browser, with the RGI glacier outlines in red and **(right)** the relative median glacier elevations. The black triangles denote some of the regions highest peaks: (1) Mount Logan, (2) Mount Saint Elias, (3) Mount Lucania, (4) King Peak, (5) Mount Bona, (6) Mount Steele, (7) Mount Wood, (8) Mount Vancouver, (9) Mount Slaggard, (10) Mount Hubbard, (11) Mount Bear, (12) Mount Walsh, (13) University Peak, and (14) Mount Augusta. Median glacier elevations do not seem to be correlated with distance from high mountains, but a dependence on the distance from the ocean is evident. There are notable anomalies associated with debris-covered glaciers, e.g., the glacier south of (2), and calving glaciers, e.g., the glacier south of (8). The base map is a shaded relief of the ASTER GDEM V003 [S1].

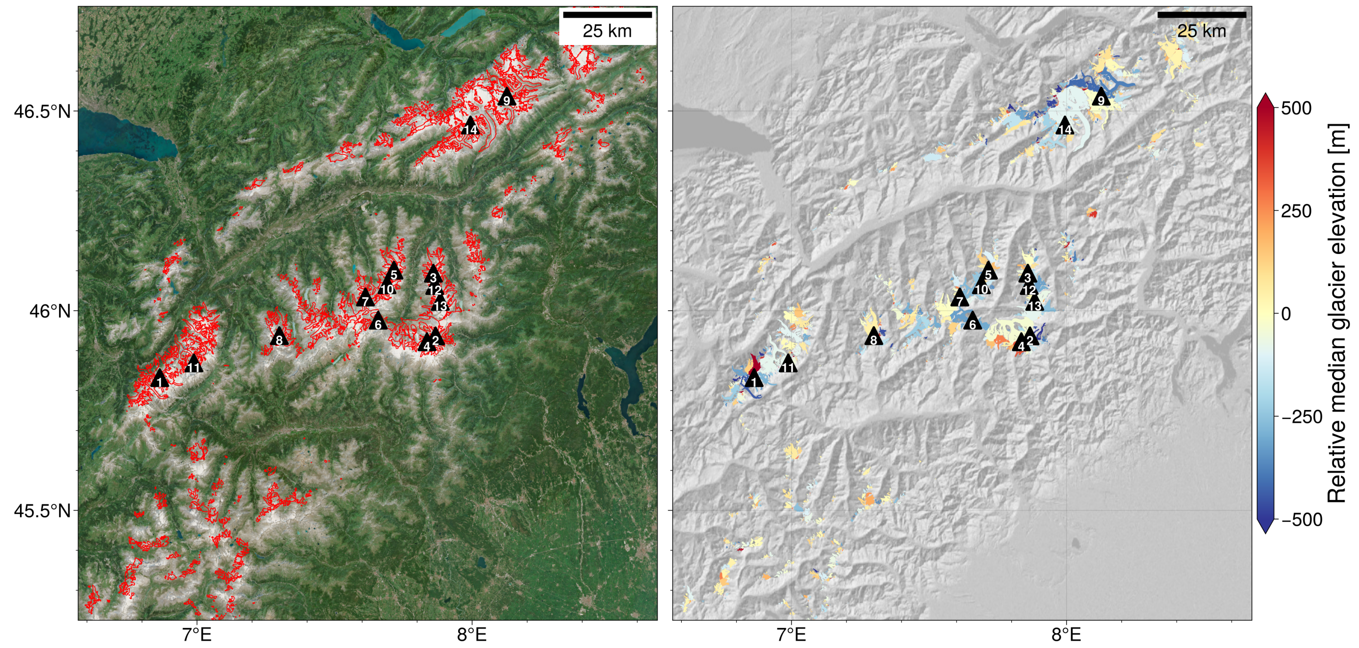

Fig. S3 The Alps: **(left)** true colour imagery from modified Copernicus Sentinel data [2024] processed in Copernicus Browser, with the RGI glacier outlines in red and **(right)** the relative median glacier elevations. The black triangles denote some of the regions highest peaks: (1) Mont Blanc, (2) Monte Rosa, (3) Dom, (4) Lyskamm, (5) Weisshorn, (6) Matterhorn, (7) Dent Blanche, (8) Grand Combin, (9) Finsteraarhorn, (10) Zinalrothorn, (11) Grandes Jorasses, (12) Alphubel, (13) Rimpfischhorn, and (14) Aletschhorn. Median glacier elevations do not seem to be correlated with distance from high mountains, but a dependence on aspect is visible, e.g., around (4). The base map is a shaded relief of the ASTER GDEM V003 [S1].

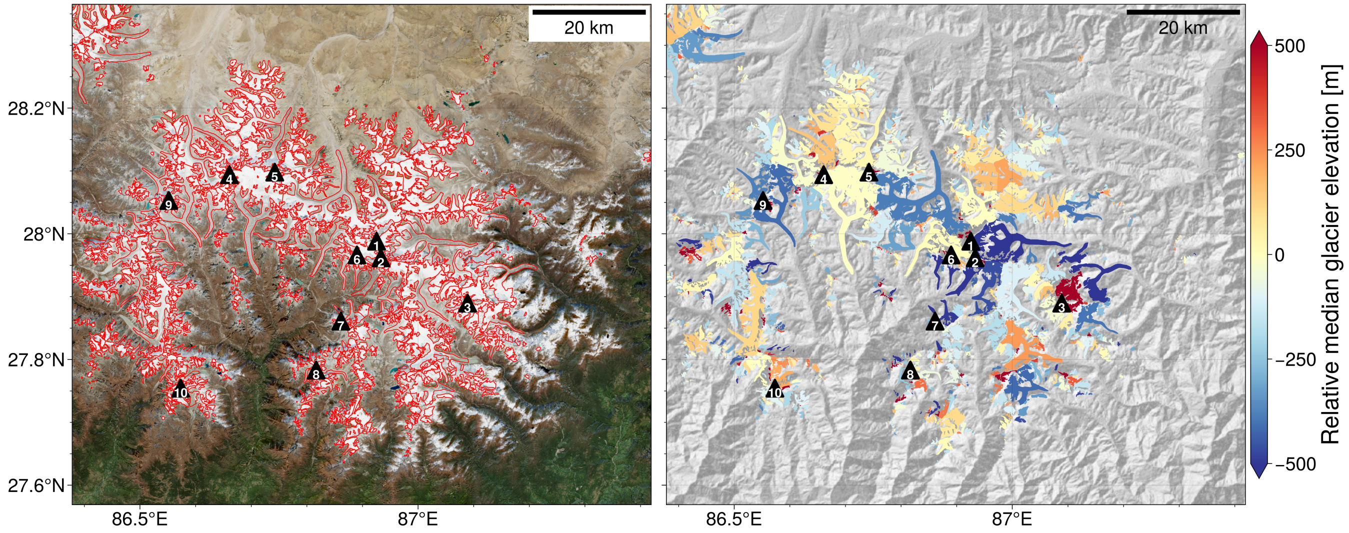

Fig. S4 The region around Mount Everest: **(left)** true colour imagery from modified Copernicus Sentinel data [2023] processed in Copernicus Browser, with the RGI glacier outlines in red and **(right)** the relative median glacier elevations. The black triangles denote some of the regions highest peaks: (1) Everest, (2) Lhotse, (3) Makalu, (4) Cho Oyu, (5) Gyachung Kang, (6) Nuptse, (7) Ama Dablam, (8) Kangtega, (9) Lunag Ri, and (10) Numbur. Median glacier elevations do not seem to be correlated with distance from high mountains, but anomalies associated with dry-calving and debris cover are observed, e.g., on the north-eastern flank of (3). The base map is a shaded relief of the ASTER GDEM V003 [S1].

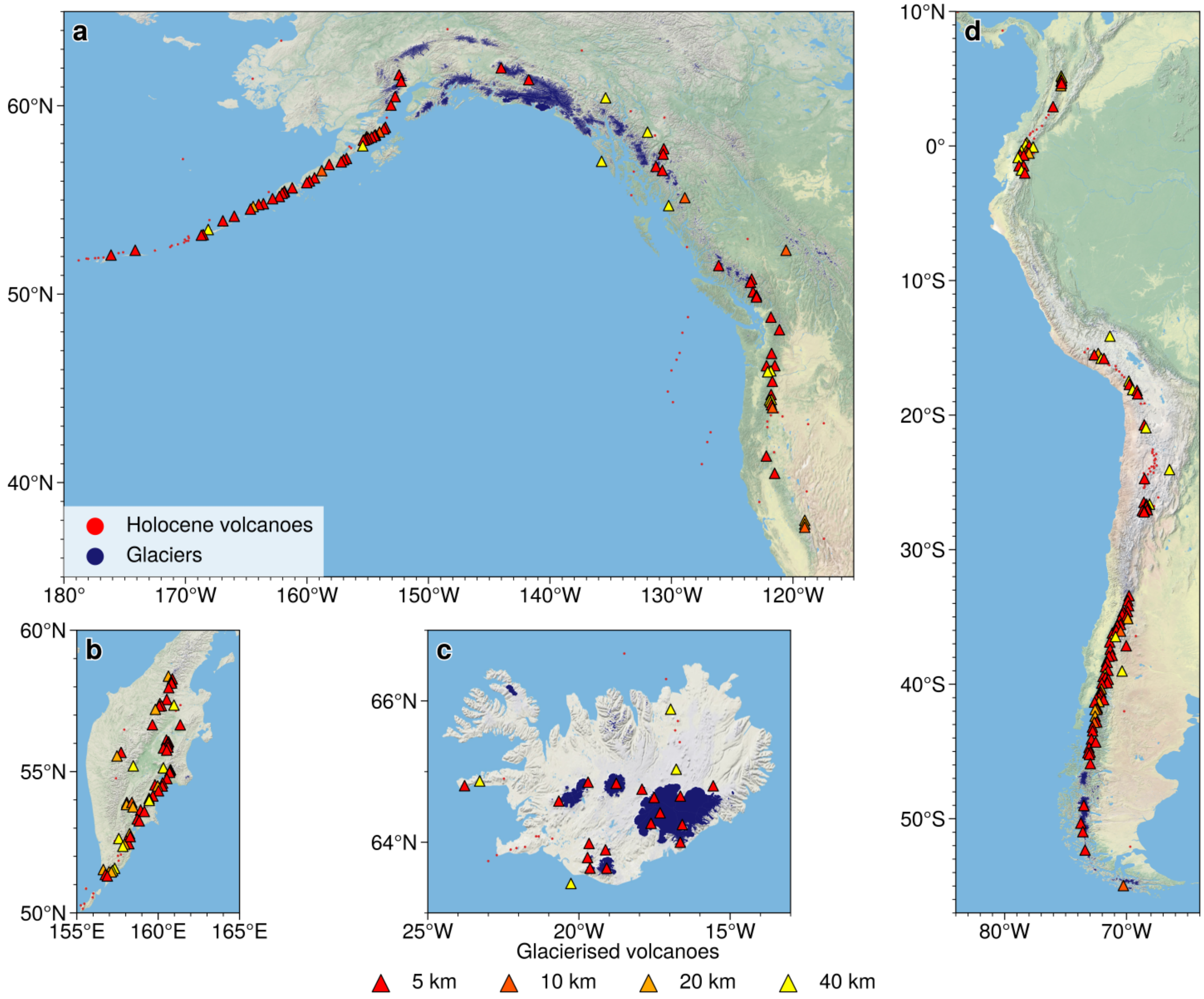

Fig. S5 Enlarged insets of Fig. 5, showing Holocene volcanoes (red dots) from the Global Volcanism Program [S2], and glaciers (blue polygons) from the Randolph Glacier Inventory [S3], for (**a**) North America; (**b**) Kamchatka; (**c**) Iceland; and (**d**) South America. The glacierised volcanoes analysed in this study (triangles) are volcanoes with glaciers within a distance of 5 km (red), 10 km (dark orange), 20 km (light orange), and 40 km (yellow). The basemaps were made with Natural Earth (naturalearthdata.com).

References

[S1] NASA/METI/AIST/Japan Spacesystems And U.S./Japan ASTER Science Team (2019). ASTER Global Digital Elevation Model V003 (NASA EOSDIS Land Processes Distributed Active Archive Center, 2019), https://doi.org/https://doi.org/10.5067/ASTER/ASTGTM.003.

[S2] GVP (2023). Volcanoes of the World, v.5.1.0 (Smithsonian Institution – Global Volcanism Program, 2023), https://doi.org/10.5479/si.GVP.VOTW5-2023.5.1, [Accessed 19 Oct 2023].

[S3] RGI Consortium (2023). Randolph Glacier Inventory - a dataset of global glacier outlines, version 7 (NASA National Snow and Ice Data Center Distributed Active Archive Center, 2023), https://doi.org/10.5067/f6jmovy5navz, [Accessed 27 Oct 2023].
